# High-Resolution Microfluidic Single-Cell Transcriptional Profiling Reveals Clinically Relevant Subtypes among Human Stem Cell Populations Commonly Utilized in Cell-Based Therapies

**DOI:** 10.3389/fneur.2016.00041

**Published:** 2016-03-22

**Authors:** Robert C. Rennert, Richard Schäfer, Tonya Bliss, Michael Januszyk, Michael Sorkin, Achal S. Achrol, Melanie Rodrigues, Zeshaan N. Maan, Torsten Kluba, Gary K. Steinberg, Geoffrey C. Gurtner

**Affiliations:** ^1^Department of Surgery, Stanford University School of Medicine, Stanford, CA, USA; ^2^Department of Neurosurgery, Stanford University School of Medicine, Stanford, CA, USA; ^3^Department of Orthopedics, University Hospital Tübingen, Tübingen, Germany

**Keywords:** single-cell analysis, stem cell therapeutics, cellular heterogeneity

## Abstract

Stem cell therapies can promote neural repair and regeneration, yet controversy regarding optimal cell source and mechanism of action has slowed clinical translation, potentially due to undefined cellular heterogeneity. Single-cell resolution is needed to identify clinically relevant subpopulations with the highest therapeutic relevance. We combine single-cell microfluidic analysis with advanced computational modeling to study for the first time two common sources for cell-based therapies, human NSCs and MSCs. This methodology has the potential to logically inform cell source decisions for any clinical application.

## Introduction

The central nervous system is uniquely susceptible to injury and possesses a limited capacity for regeneration. Stem and progenitor cells are a promising therapeutic option as they potentially provide both cytokines and cellular substrate to promote tissue survival and regeneration. As such, cell therapies from a variety of sources [e.g., adult multipotent bone marrow (BM) and neural stem cells, and embryonic or induced pluripotent/neural progenitor cells] have been explored for a wide range of neurologic disorders, including Parkinson’s disease, stroke, and spinal cord injury (1, 2). Despite pre-clinical efficacy, there has been delayed clinical translation of this work as the mechanistic hypothesis has largely shifted from neural replacement to enhancing endogenous repair processes (3, 4). Recent increases in our understanding of the heterogeneity of stem and progenitor cell populations (5–8) provide a potential explanation for variable stem cell therapeutic efficacy, while also presenting an opportunity to tailor cell-based approaches to specific clinical applications.

Until recently, analytic approaches possessed inadequate resolution to study heterogeneous samples, such as stem and progenitor cells (9, 10), because the pooling of nucleic acids or proteins from hundreds of thousands of cells analyzed in aggregate is unable to account for cellular heterogeneity and potentially distinct cell subgroups. However, advances in microfluidic technology have enabled massively parallel single-cell gene expression analyses that for the first time permit the high-resolution study of cell subpopulation heterogeneity and complex intercellular interactions (11–13). Leveraging this technology, we have refined a platform capable of performing high-resolution, high-throughput analyses of therapeutic and other cell sources (14–20). This approach combines single-cell transcriptional interrogation with advanced computational statistics to visualize cellular heterogeneity and identify distinct subpopulations for prospective enrichment. Characteristic cell signaling pathways can also be identified and compared, thus providing a logical approach to cell source-application matching in the clinical setting.

## Methods

### Human Bone Marrow Mesenchymal Stem Cell Isolation and Culture

Human bone marrow mesenchymal stem cells (hBM-MSCs) were isolated and cultured, as described previously (7, 21). Briefly, following written informed consent and approval of the ethical committee of the University Hospital Tübingen, Germany, BM aspirates from adult patients were obtained during orthopedic operations. BM mononuclear cells were isolated by density gradient centrifugation, washed twice with phosphate buffered saline (PBS, Lonza, Walkersville, MD, USA), and seeded at a density of 1 × 10^5^ cells/cm^2^ in culture medium is composed of alpha minimum essential media (α-MEM, Lonza), 1% penicillin–streptomycin (Lonza), and 10% pooled human blood group AB serum (ZKT Tübingen, Germany). Freshly obtained (P0) hBM-MSCs were cultured under standard conditions (37°C, 5% CO_2_), with non-adherent cells removed after 24 h. Medium was changed twice a week until cells reached subconfluency. hBM-MSCs were detached using Trypsin-EDTA (Lonza), counted using a CASY^®^ cell counter (Roche, Basel, Switzerland), and cryopreserved for shipping to the United States. Upon thawing, hBM-MSCs from five separate donors were pooled and plated at a density of 1 × 10^4^ cells/cm^2^ for the next passage (P1). Cultured P1 hBM-MSCs were analyzed by microfluidic single-cell transcriptional profiling. Cultured P2 hBM-MSCs were analyzed by flow cytometry.

### Human Neural Stem Cell Derivation and Culture

Human neural stem cells (hNSCs) were generated from human embryonic stem cells (hESCs), as previously described (22). Briefly, to generate hNSCs, dissociated hESCs [from the H9 cell line (WiCell Research Institute, Madison, WI, USA)] were cultured in medium composed of Dulbecco’s modified Eagle’s medium (DMEM) and F12 nutrient (1:1 ratio), supplemented with glucose (0.6%), glutamine (2 mM), sodium bicarbonate (3 mM), and HEPES buffer (5 mM) [all from Sigma-Aldrich (St Louis, MO, USA) except glutamine (Invitrogen Life Technologies, Grand Island, NY, USA)]. A hormone and salt mixture (Sigma), composed of insulin (25 mg/ml), transferrin (100 mg/ml), progesterone (20 nM), putrescine (60 mM), and selenium chloride (30 nM), was used in place of serum, and the medium was also supplemented with epidermal growth factor (EGF, 20 ng/ml), basic fibroblast growth factor (bFGF, 10 ng/ml), and leukemia inhibitory growth factor (LIF, 10 ng/ml). Cells were initially seeded at a density of 1 × 10^5^ cells/ml in Corning T75 (Invitrogen) culture flask, and after 5–7 days (prior to reaching confluency) the adherent culture was incubated in 0.025% trypsin/0.01% EDTA (w/v) for 1 min, followed by the addition of trypsin inhibitor (Invitrogen) then gently triturated to achieve single cell suspension. The cells were then washed twice with fresh medium and reseeded in fresh growth factor-containing media at 1 × 10^5^ cells/ml. Subconfluent hNSCs were serially expanded *in vitro* prior to single cell and flow cytometric analyses.

### Flow Cytometry and Microfluidic Single-Cell Gene Expression Analysis

Single-cell reverse transcription and low cycle pre-amplification were performed, as previously described (15). Briefly, following 12 h of serum starvation to synchronize cell cycles, cell suspensions of hBM-MSCs and hNSCs were sorted as single cells into each well of a 96-well plate using a FACSAria flow cytometer (BD Biosciences, San Jose, CA, USA) into 6 μl of lysis buffer and SUPERase-In RNAse inhibitor (Applied Biosystems, Foster City, CA, USA). Live/dead gating was performed based on propidium iodide exclusion. Reverse transcription and low-cycle pre-amplification was performed following addition of Superscript III reverse transcriptase enzyme (Invitrogen, Carlsbad, CA, USA), Cells Direct reaction mix (Invitrogen, Carlsbad, CA, USA), and target gene-specific TaqMan assay (primer/probe) sets (Applied Biosystems) (Tables S1 and S2 in Supplementary Material) [20 min at 50°C, 2 min at 95°C, followed by a gene target-specific 22-cycle pre-amplification (denature at 95°C for 15 min, anneal at 60°C for 4 min, each cycle)]. Exon-spanning primers were used where possible to avoid amplification of genomic background. Resultant single-cell cDNA was mixed with sample loading agent (Fluidigm, South San Francisco, CA, USA) and Universal PCR Master Mix (Applied Biosystems) and loaded into 96.96 Dynamic Array chips (Fluidigm) along with TaqMan assays (Tables S1 and S2 in Supplementary Material) and assay loading agent according to the manufacturer’s instructions (Fluidigm). Products were analyzed on the BioMark reader system (Fluidigm) using a hot start protocol to minimize primer-dimer formation, 40 quantitative PCR cycles were performed. Gene targets were selected after an exhaustive literature review relating to cell stemness, vasculogenesis, and neuronal regeneration for hBM-MSC analyses, and to cell stemness and lineage differentiation for hNSC analyses. Selected cell surface markers, housekeeping, and control genes were included in all microfluidic runs.

Flow cytometry was performed according to manufacturer’s instructions on a FACSAria flow cytometer (BD Biosciences). Briefly, hBM-MSCs and hNPCs cultured as above were incubated for 20 min in FACS buffer (PBS supplemented with 2% FBS) containing anti-human PE-conjugated TFRC [hBM-MSCs (BD Biosciences)], PE-conjugated PROM1 [hNSCs (Miltenyi Biotec, San Diego, CA, USA)] or PE-Cy7-conjugated CCR4 [hNScs (Biolegend, San Diego, CA, USA)] antibodies, respectively, and washed thoroughly prior to analysis.

### Statistical Analysis

Analysis of single-cell data was performed, as described previously (14, 15). The goal of this analysis was to identify cell subpopulations with similar transcriptional signatures within putatively homogeneous populations (e.g., hBM-MSCs and hNSCs). Briefly, expression data from experimental chips were normalized relative to the median expression for each gene in the pooled sample and converted to base 2 logarithms. Absolute bounds (±5 cycle thresholds from the median, corresponding to 32-fold increases/decreases in expression) were set, and non-expressers were assigned to this floor. Clustergrams were then generated using hierarchical clustering (with a “complete” linkage function and Euclidean distance metric) in order to facilitate data visualization via MATLAB (R2011b, MathWorks, Natick, MA, USA).

To detect overlapping patterns within the single-cell transcriptional data, k-means clustering was employed using a standard Euclidean distance metric. Accordingly, each cell was assigned membership to a specific cluster as dictated by similarities in expression profiles (minimizing the within-cluster sum of square distances) in MATLAB. Optimally partitioned clusters were then sub-grouped using hierarchical clustering to facilitate visualization of data patterning (15). Partitional clustering of hNSCs for Figure S4 in Supplementary Material was achieved through limiting our k-means algorithm to a subset of genes classified as “secreted factors,” whereas all 96 genes were utilized for purposes of gene-wise and intra-cluster cell-wise hierarchical clustering. In all single-cell data representations, gene-wise hierarchical clustering is visualized on the left, while cell-wise hierarchical clustering is on top.

Non-parametric, two-sample Kolmogorov–Smirnov (K–S) tests were used to identify those genes with expression patterns that differed significantly between population clusters and/or groups, following Bonferroni correction for multiple samples using a strict cutoff of *p* < 0.05. For subgroup comparisons, the empirical distribution of cells from each cluster was evaluated against that of the remaining cells in the experiment.

Ingenuity Pathway Analysis (IPA, Ingenuity Systems, Redwood City, CA, USA) was used to construct transcriptome networks based on genes that were significantly increased between hBM-MSCs and hNSCs. For this analysis, the 68 common genes included in the corresponding single-cell analyses (rather than the entire transcriptome) was used as the reference set, in order to avoid biasing the associated enrichment calculations in IPA’s internal network generation algorithm.

## Results and Discussion

**Figure 1:**
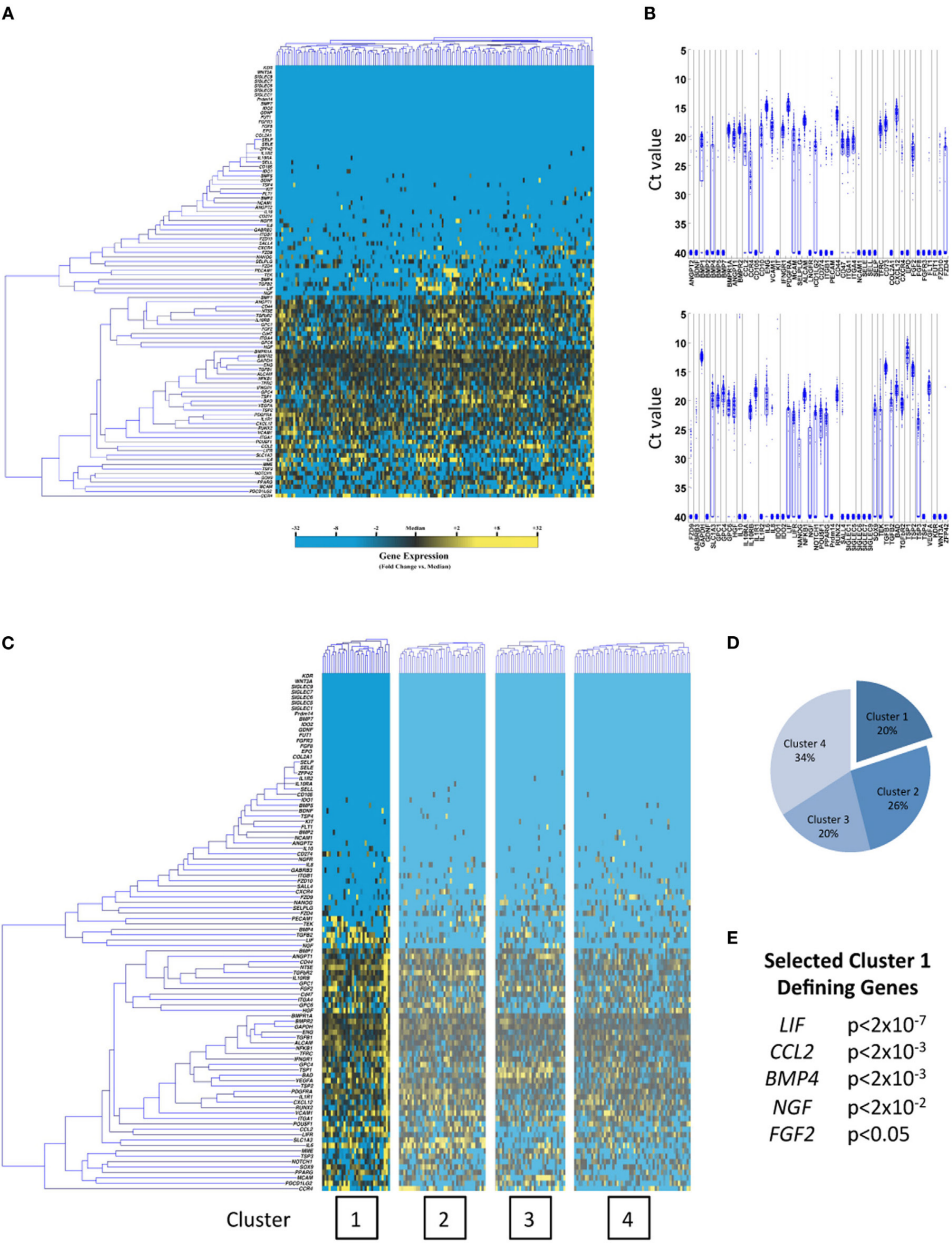
**Single-cell transcriptional analysis of hBM-MSCs**. **(A)** Hierarchical clustering of cells (gene-wise clustering on left, cell-wise clustering on top). Gene expression presented as fold change from median (yellow, high expression, 32-fold above median to blue, low expression, 32-fold below median). **(B)** Whisker plots presenting raw qPCR cycle threshold values for each gene across all cells. Individual dots represent single gene/cell qPCRs, with increased cycle threshold values corresponding to decreased mRNA content. Cycle threshold values of 40 represent failed amplifications. **(C)** K-means clustering of hBM-MSCs (*k* = 4). **(D,E)** hBM-MSC cluster pie chart representing the fraction of cells comprising each cluster and selected cluster 1 defining genes determined via Kolmogorov–Smirnov testing.

**Figure 2:**
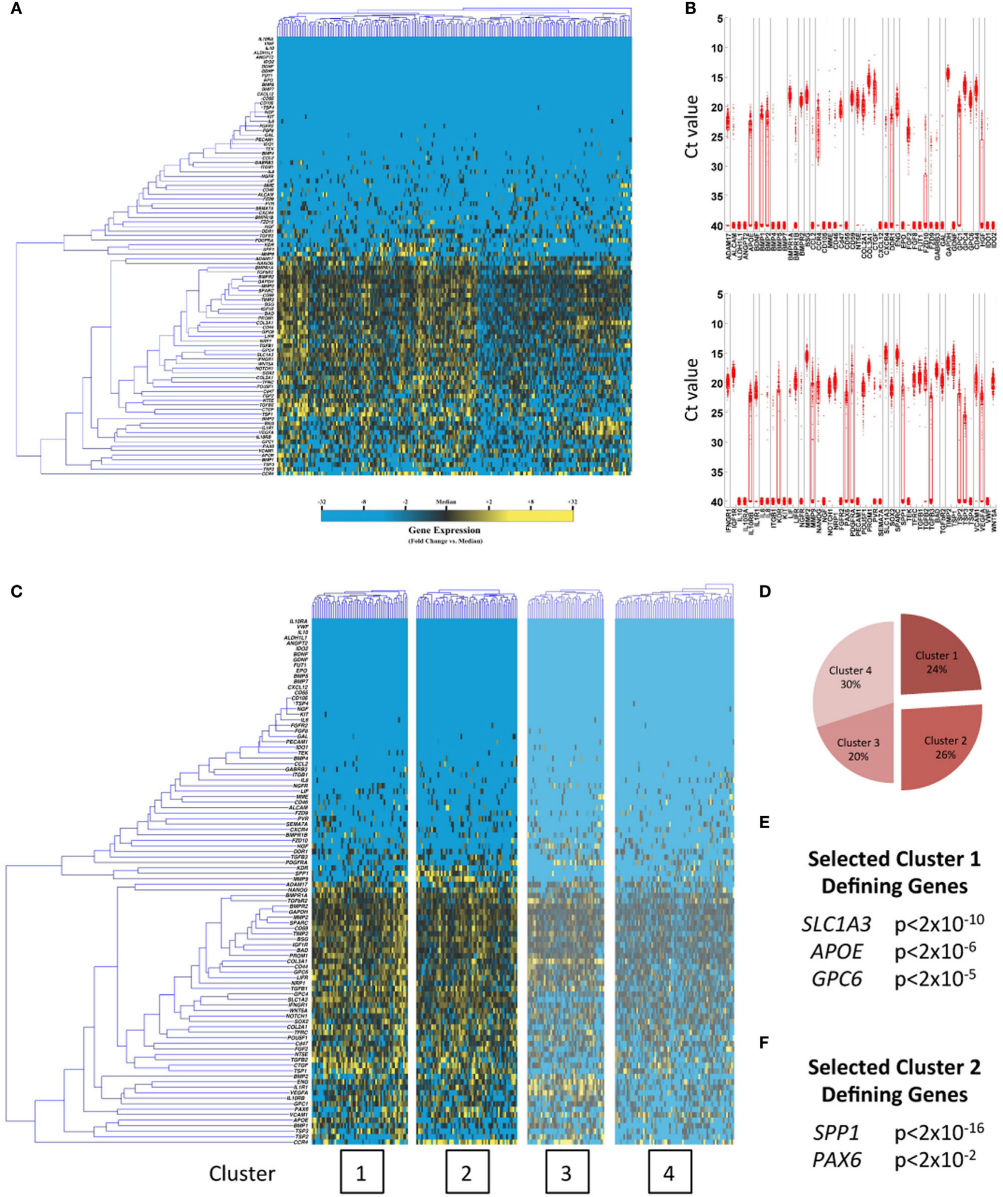
**Single-cell transcriptional analysis of hNSCs**. **(A)** Hierarchical clustering, whisker plots **(B)**, and K-means clustering **(C)** of hNSCs (*k* = 4). **(D–F)** hNSC cluster pie chart representing the fraction of cells comprising each cluster and selected cluster 1 and 2 defining genes determined via Kolmogorov–Smirnov testing.

In this work, we for the first time characterize subpopulations in hBM-MSCs and hESC-derived neural stem cells (hNSCs), utilizing this microfluidic single-cell approach, to gain insights into the optimal clinical applications of these cell sources. Interestingly, significant heterogeneity was observed in both cell types (Figures 1 and 2A,B). Moreover, automated partitional clustering (i.e., cell groupings based on similarities in gene expression; see “Statistical Analysis” in Section “Methods” for complete description) of these data identified distinct transcriptionally defined cellular subpopulations of clinical relevance (Figures 1 and 2C,D), with gene expression profiles suggestive of a potentially beneficial effect of subfractionation. In particular, a distinct subpopulation of hBM-MSCs displayed enhanced expression of genes encoding secreted factors associated with neuronal growth, differentiation, and survival (such as *LIF*, *CCL2*, *BMP4*, *NGF*, and *FGF2*) (Figures 1C–E), making it particularly appealing for neuroregenerative cell therapy applications, such as following ischemic or traumatic insult. Conversely, hNSCs possessed two cell subpopulations with gene profiles suggestive of differential lineage commitment [i.e., pre-astrocytes/glial cells defined by *SLC1A3*, *APOE*, and *GPC6* expression (23–25) and pre-neurons characterized by *SPP1* and *PAX6* (26, 27)] (Figures 2C–F), further supporting the concept of functional cell heterogeneity within precursor cell populations and highlighting the potential for targeted purification based on clinical need. Importantly, the subpopulations of interest in both hBM-MSCs and hNSCs were co-defined by expression of cell surface marker genes (Figures S1 and S2 in Supplementary Material), which may enable their prospective isolation for experimental or therapeutic application.

**Figure 3:**
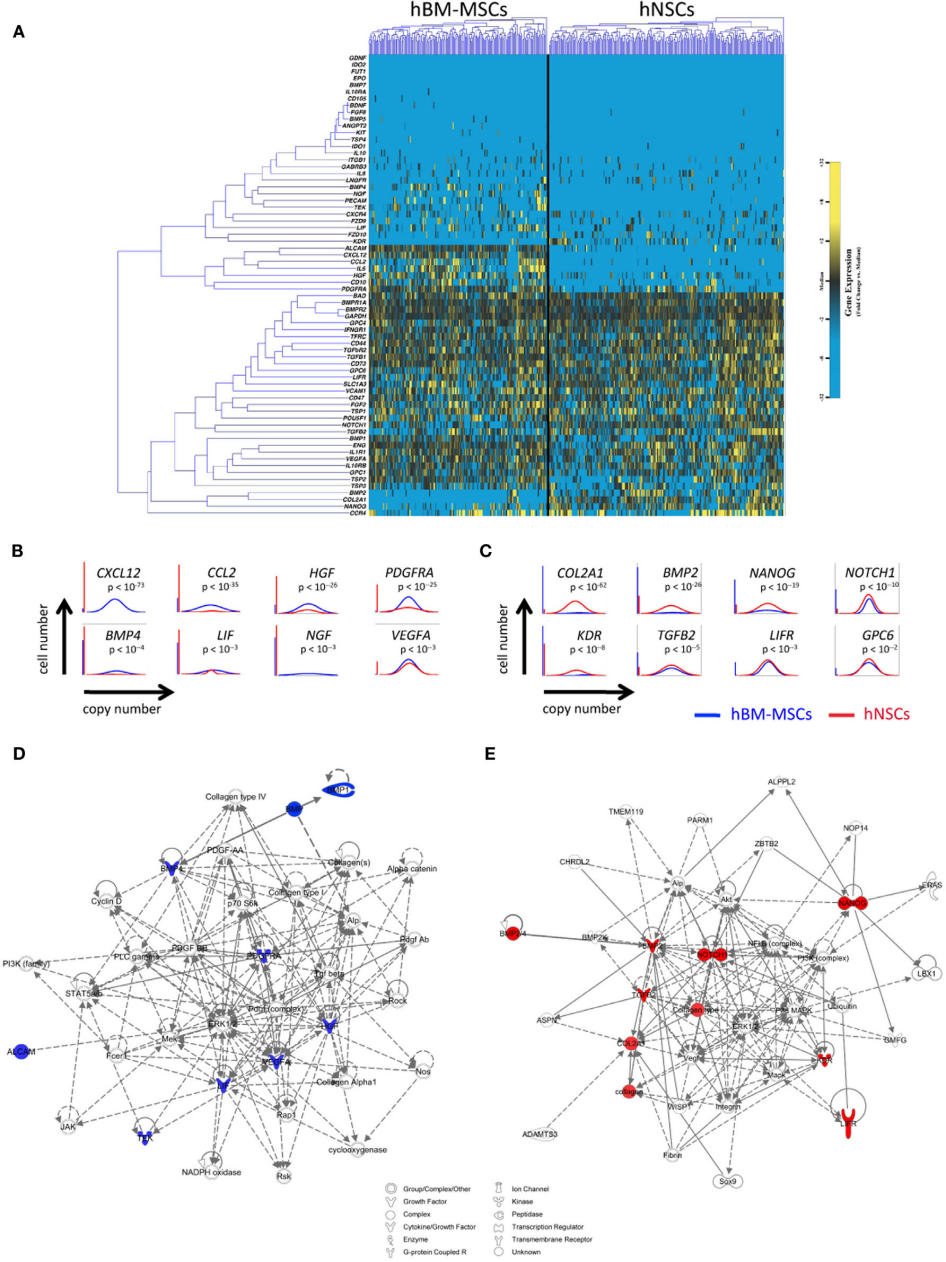
**Comparative single-cell analysis of hBM-MSCs and hNSCs**. **(A)** Hierarchical clustering of cells from hBM-MSCs (left) and hNSCs (right) with gene expression presented as fold change from median. **(B,C)** Selected differentially expressed genes relating to cell stemness and pro-vascular/neuronal survival between hBM-MSCs and hNSCs identified using non-parametric two-sample Kolmogorov–Smirnov testing, illustrated with median-centered Gaussian curve fits [**(B)** genes upregulated in hBM-MSCs; **(C)** genes upregulated in hNSCs]. The left bar for each panel represents the fraction of qPCRs that failed to amplify in each group. **(D,E)** Top scoring Ingenuity Pathway Analysis (IPA)-constructed transcriptome networks based on genes significantly increased in hBM-MSCs **(D)** and hNSCs **(E)**, respectively. Significant “seed” genes are colored in blue or red to distinguish them from the remaining “inferred” entities in the network.

Despite likely differences in mechanism of action, progenitor cells from disparate sources have been tested in similar neuroregenerative settings. To gain insight into the signaling differences across cell types that may guide their clinical application, we directly compared the single-cell transcriptional profiles and activated gene networks of hBM-MSCs and hNSCs (Figure 3). Consistent with the developmentally distinct origins of these cells (i.e., adult versus embryonic derived), significant differences were observed across cell types in this analysis, although each displayed a pro-regenerative profile. Specifically, hBM-MSCs possessed a pro-vascular phenotype (defined in part by expression of *CXCL12*, *PDGFRA*, *VEGFA*, and *HGF*) (Figures 3B,D), while hNSCs featured a more stem-like and pro-neuronal profile (including upregulation of *BMP2*, *NANOG*, and *GPC6*) (Figures 3C,E). Subclustering of these combined single-cell data suggests that these cell entities would function differently following injury, with cell origin being the dominant driver of cluster formation. In fact, there was an absence of common, transcriptionally defined cellular subpopulations across groups (Figure S3 in Supplementary Material). Importantly, the identification of such cell-specific signaling suggests a potential synergy of mixed cell therapies for clinical applications. For example, LIF expression in hBM-MSCs (a cytokine that promotes neuronal precursor differentiation) (28) could be coupled with LIF receptor (LIFR) expression in hNSCs to simultaneously promote neovascularization and site-specific differentiation of applied neural precursors.

These data demonstrate the utility of single-cell analysis for the characterization and potential improvement of cell-based therapeutics for neurodegenerative and other diseases. Moreover, the heterogeneity of the cell populations studied herein, only visible with this or similar resolution platforms, highlights the potential for tailoring cell therapies based on clinical need. Specifically relating to stroke therapies, the recently proven efficacy of endovascular thrombectomy for large vessel occlusions (29–32) provides a currently unutilized opportunity to deliver neuroprotective and regenerative cells directly to ischemic brain tissue upon revascularization. In this scenario, single or combined cell subpopulations with desired cell profiles could be prospectively isolated prior to application. Although unbiased partitional clustering was used herein to identify physiological cell subpopulations, an alternative analytical approach with restricted clustering based on desired gene expression (such as secreted cytokines and growth factors) could be employed to prospectively identify groups of cells with a specific profile of interest (Figure S4 in Supplementary Material). As such, we envision that a similar methodology could be applied to any cell type to logically inform cell source decisions and improve cell-based therapies for neurologic pathologies.

## Author Contributions

All listed authors contributed to the idea generation, design, and completion of this work. RR, RS, and TB contributed equally to the idea generation, experimental work, and manuscript preparation. MJ, MS, AA, MS, ZM, and TK contributed to the experimental work, manuscript preparation, and revisions. GS and GG guided the idea generation, experimental work, and manuscript preparation.

## Conflict of Interest Statement

The authors declare that the research was conducted in the absence of any commercial or financial relationships that could be construed as a potential conflict of interest. The reviewer CS and handling Editor declared their shared affiliation, and the handling Editor states that the process nevertheless met the standards of a fair and objective review.

## Supplementary Material

Figure S1 **List of hBM-MSC and hNSC cluster defining genes determined via Kolmogorov–Smirnov testing from Figures 1 and 2. Bold genes encode cell surface markers**.

Figure S2 **(A)** Flow cytometric analysis of hBM-MSCs demonstrating differential expression of the cell surface protein TFRC, a potential marker for prospective hBM-MSC Cluster 1 isolation. **(B)** Flow cytometric analysis of hNSCs demonstrating differential expression of the cell surface proteins PROM1 and CCR4 (hi/low gating), potential markers for hNSC Cluster 1 and 2 enrichment, respectively. Left plots negative controls and right plots stained cells.

Figure S3 **(A)** Hierarchical clustering of cells from hBM-MSCs (left) and hNSCs (right) with gene expression presented as fold change from median (as presented in **Figure 3**). **(B)** K-means clustering of hBM-MSCs and hNSCs (*k* = 1–5), with pie charts representing the fraction of cells comprising each cluster [hBM-MSC (blue), hNSC (red)].

Figure S4 **(A,B)** Single-cell transcriptional analysis of hNSCs clustered on genes for coding for secreted proteins (*k* = 4) **(A)**. **(B)** List of secreted protein gene names with percent of cellular expression. **(C)** Pie chart representing the fraction of hNSCs comprising each cluster, with associated surface marker genes from the highest expressing cluster (cluster 1) **(D)**, which could potentially enable subpopulation enrichment.

Table S1 **Gene names and assay IDs for microfluidic single-cell gene expression analysis of hBM-MSCs**. Genes specifically relating to stemness, neuronal survival, and vasculogenesis were chosen, in addition to selected control, cell-cycle, and surface marker-related probes.

Table S2 **Gene names and assay IDs for microfluidic single-cell gene expression analysis of hNSCs**. Genes specifically relating to stemness, neuronal survival, and vasculogenesis were chosen, in addition to selected control, cell-cycle, and surface marker-related probes.
