# Supplementary figures and images for: High-Resolution Microfluidic Single-Cell Transcriptional Profiling Reveals Clinically Relevant Subtypes among Human Stem Cell Populations Commonly Utilized in Cell-Based Therapies
Source: Front Neurol. 2016 Mar 22;7:41. doi: 10.3389/fneur.2016.00041 (PMC4801858; doi:10.3389/fneur.2016.00041)

**
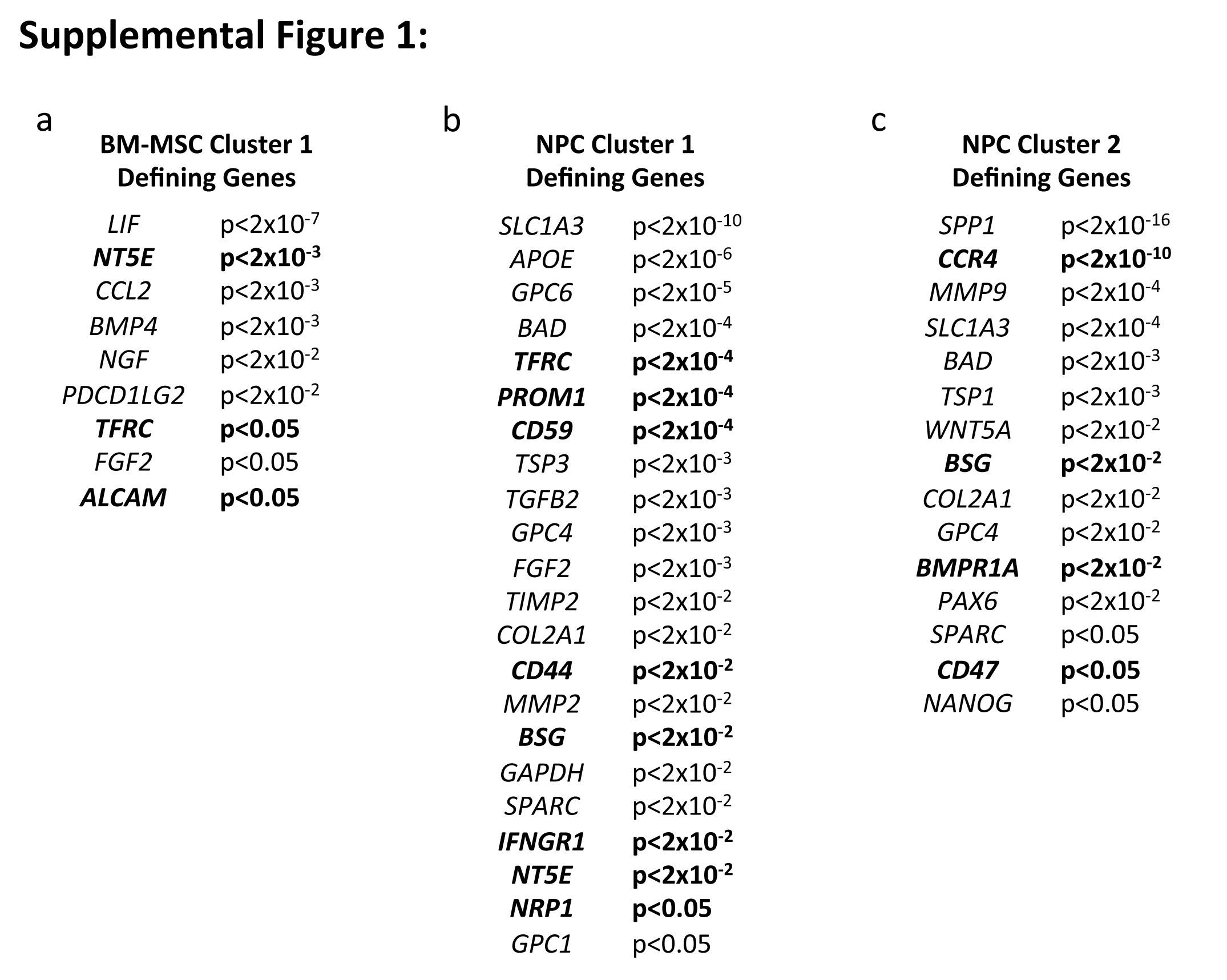
**

**
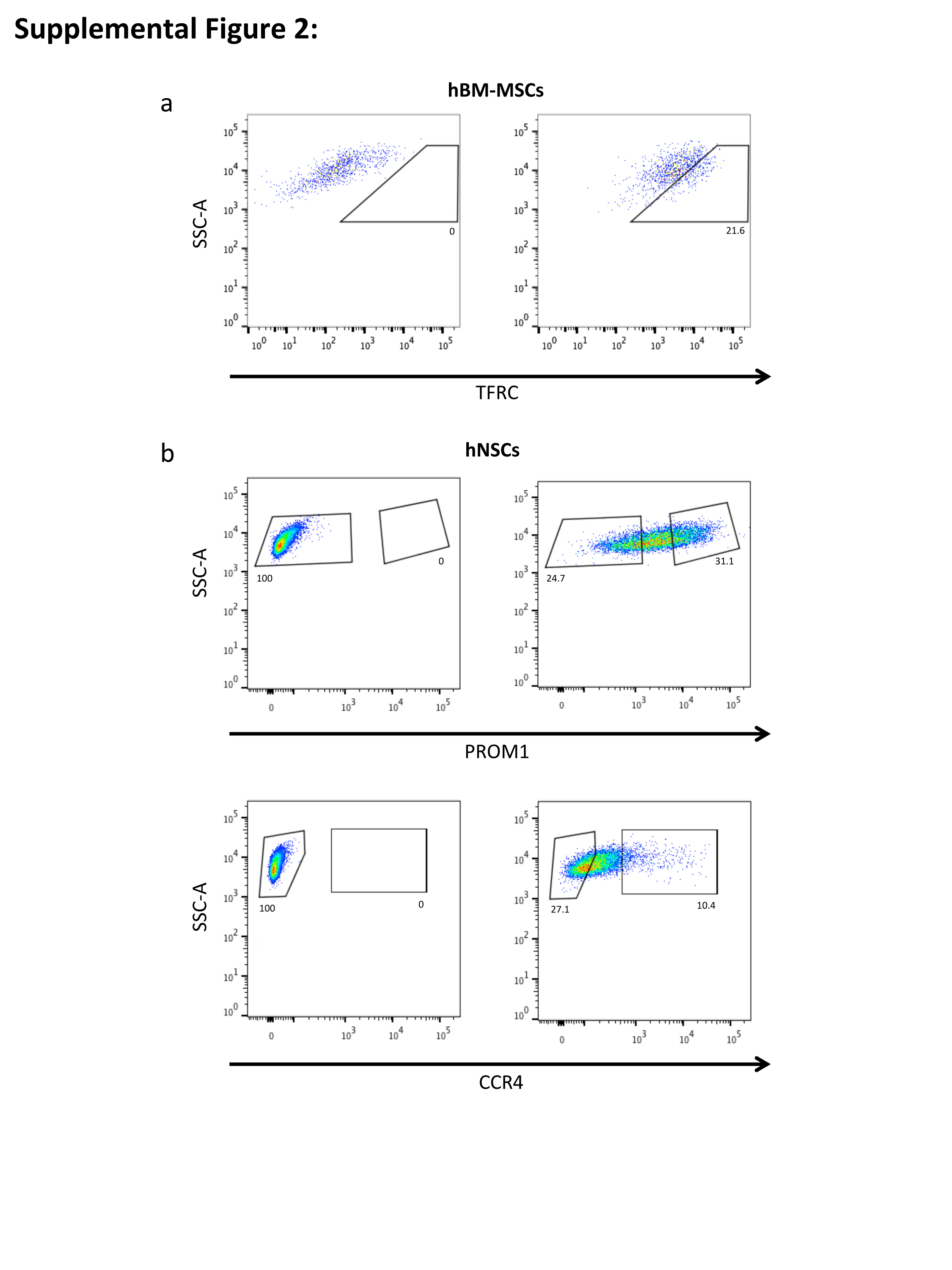

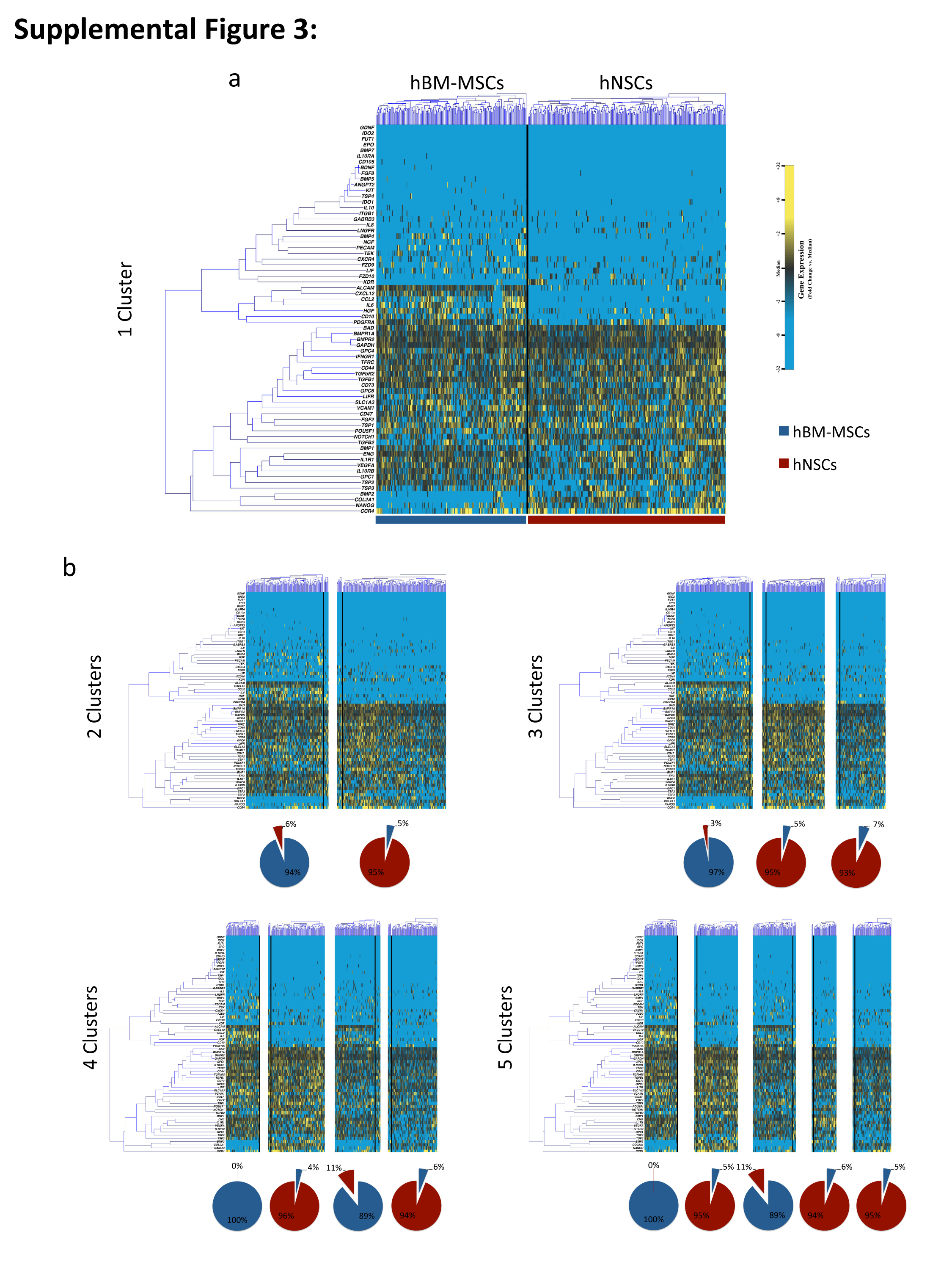

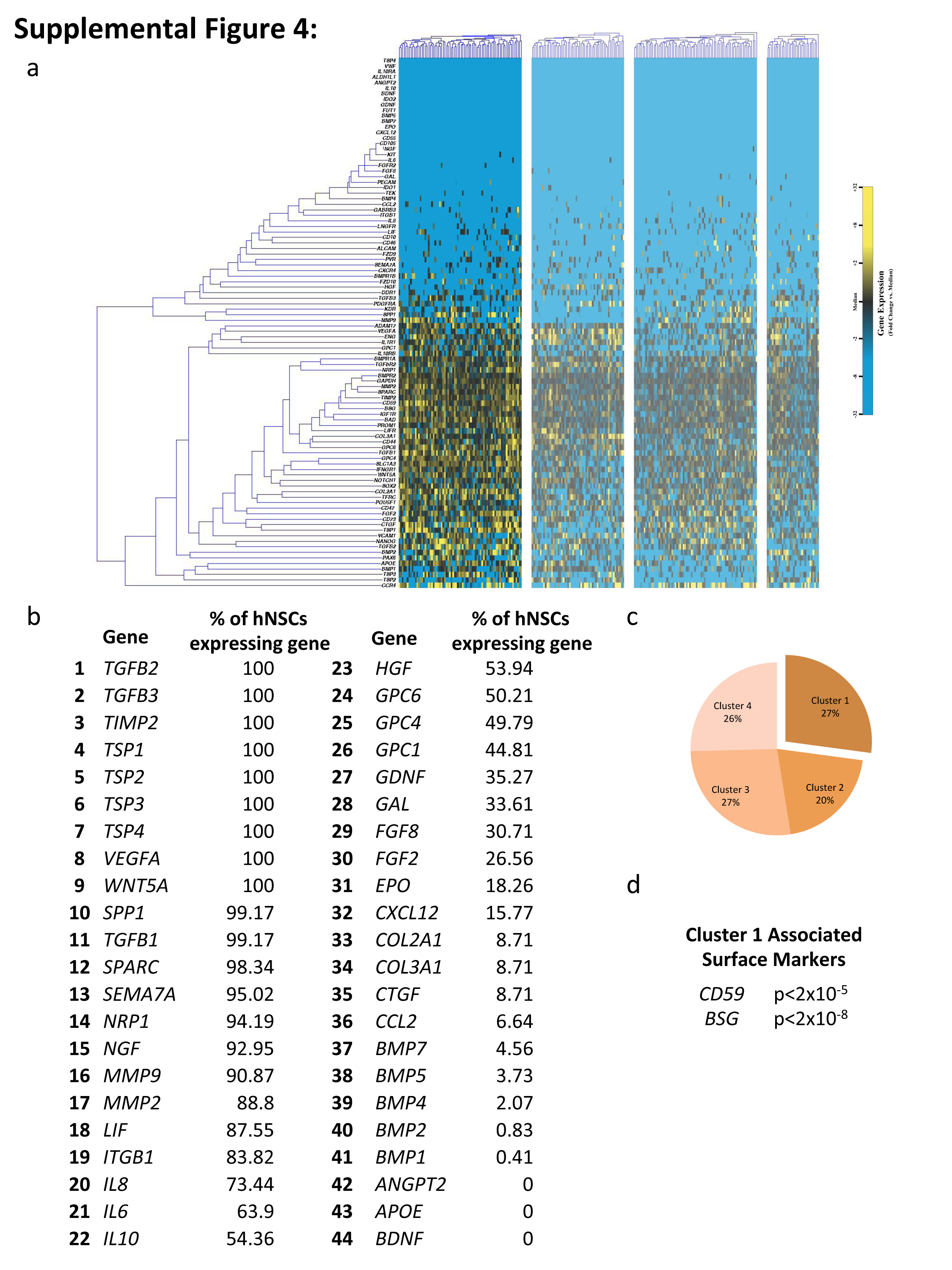

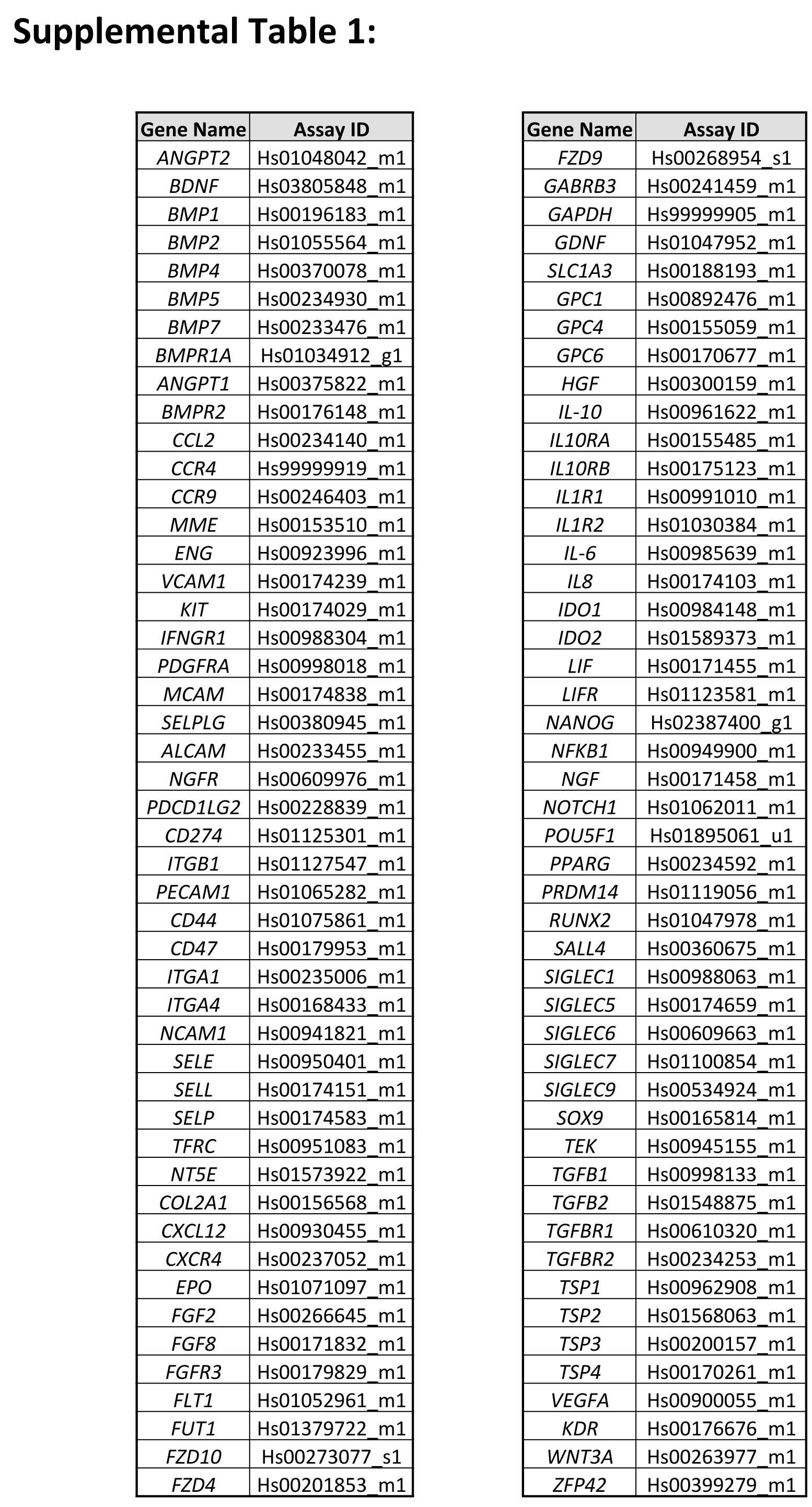
**


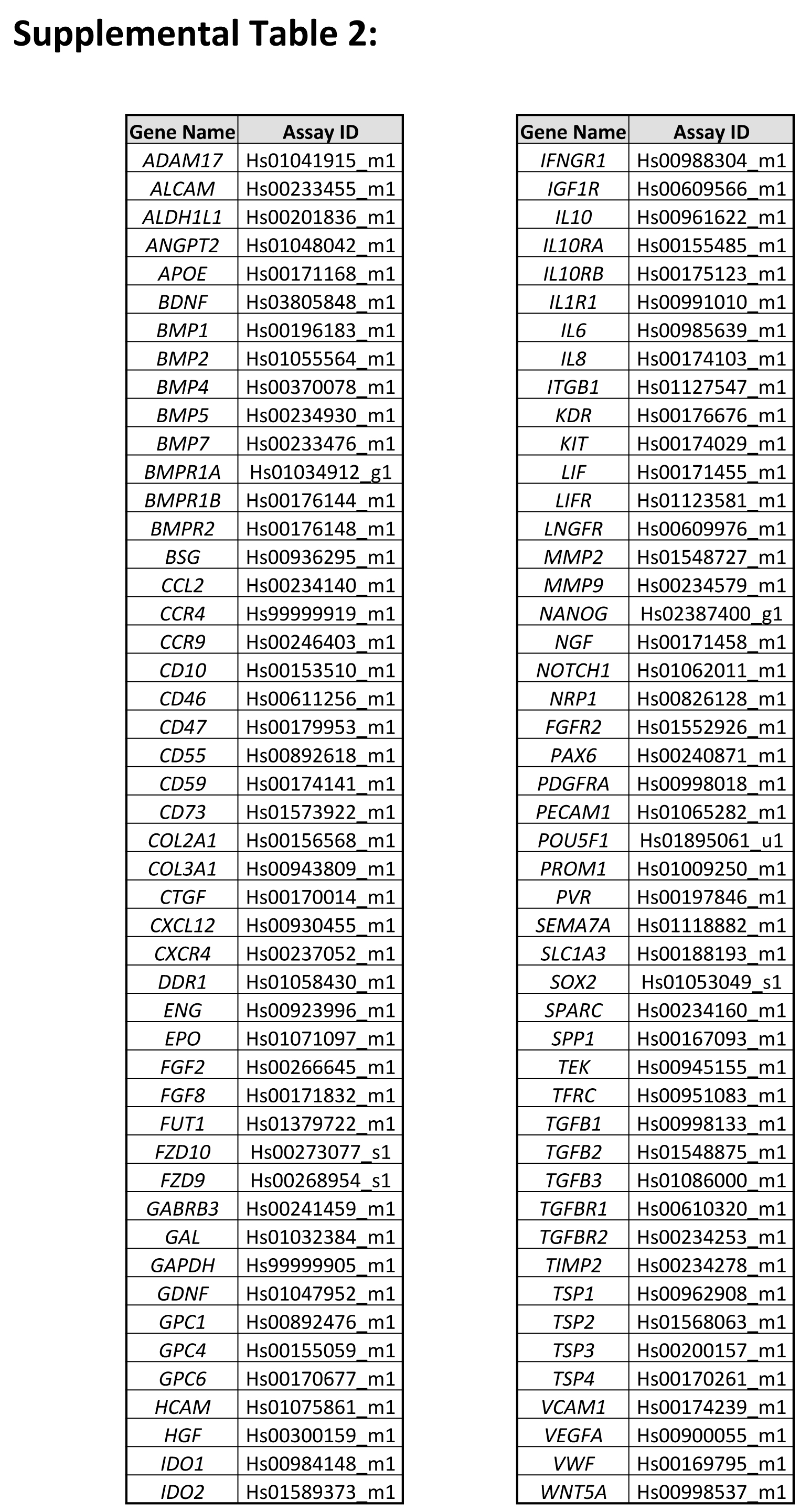

Supplement: Figure S1 — List of hBM-MSC and hNSC cluster defining genes determined via Kolmogorov–Smirnov testing from Figures 1 and 2. Bold genes encode cell surface markers. [file Data_Sheet_1.docx]
